# Homocysteine levels in septic shock

**DOI:** 10.1186/2197-425X-3-S1-A305

**Published:** 2015-10-01

**Authors:** L Mateu Campos, L Galarza Barrachina, M Arlandis Tomas, F Sanchez Morán, A Belenguer Muncharaz, D Ferrándiz Sellés, S Altaba Tena, J Torres Garcia, G Pagés Aznar

**Affiliations:** Hospital General de Castellon, Castellón de la Plana, Spain; Hospital de la Plana, Vila-real, Spain

## Objectives

To assess the role of homocysteine (HCY) in sepsis, its relationship with inflammation and its possible prothrombotic effect.

## Methods

Prospective cohort study of patients admitted to the Intensive Care Unit with criteria of septic shock (SCCC 2001), mechanically ventilated and requiring vasoactive drugs. Demographic variables, haemodynamics, respiratory, renal function, CRP and lactate were collected. HCY levels, coagulation factors II, VII, VIII and XII were determined at admission (T1), at 48 (T2) and 72 hours (T3), and at discharge from ICU or prior to death (T4) were analyzed.

## Results

Twenty-three (12 males, 11 females), mean age 74.5 years and APACHE II 18.33 ± 6.59 were included. The type of admission was surgical in 11 patients and medical in 12 patients. The overall mortality was 39% (9 patients). HCY levels were higher in those patients who died and these differences were significant at T2, T3 and at the end of the study (T4) (p < 0.005). Plasma HCY levels revealed a statistically significant positive correlation with plasma factor VII (r = 0.35, p < 0.05) and plasma factor VIII (r = 0.31, p < 0.05) at admission. Also, a statistically significant positive correlation was obtained between HCY and the cytokines IL-8, IL-10 and IL-10/ TNF-a ratio at 48 hours of the study.

## Conclusions

High HCY levels in septic patients are associated with increased mortality. HCY plays a major role in septic patients due to its pro-inflammatory and pro-coagulant effect.

